# Supplementary material for: Chromothripsis during telomere crisis is independent of NHEJ, and consistent with a replicative origin
Source: Genome Res. 2019 May;29(5):737–49. doi: 10.1101/gr.240705.118 (PMC6499312; doi:10.1101/gr.240705.118)
Supplement: Supplemental Material [file supp_gr.240705.118_Supplemental_file_1.zip › contigs/annotated_contigs/DB40/contig.2.DB40_length_555_mean_cov_9.00900900901.docx]

**DB40_length_555_mean_cov_9.00900900901**

```
AGGGAAAAAAGAAAAGAAACAACAACCATGTAGCCACTCCCTGGGGTGGGGAGGAAGGGTTAGGAGTTTAGAGTGAATCTTGGAGGAGG
 >chr2:46771483-46771886 + E=5e-231
AGGGGTTCCCCCCGGCCCTACTTCAGAAGGGCCAGGTGACTTTGTCTGGGTTTTCTTTGGGAGAGATTAATTGTGCAGAAAGAAGATGC

ACACTTTGCCCTGCAGCCTCTGGCCAGGGTTTGCACATCAGAGGTGGCCAGGCTGTGCTTCTAAAGGGCCAAGGTCTTCTCTCTGTCTC

CCTGTCCCAGCTTCAGCCTCTTCCCAAACCCACTTTTGCTCTGGGGATAAGTTGTGTGTGATGAATTTTGTAGAATCCAGCACTATGAT

TAGAGTTGAAGACTCTGGCAGAAGTTTCCACATGTCATACAGACT|TA|TATACATATACATATATATATGTATATGTATATACATATA
                                              >chr2:46773621-46773777 + E=4e-67
TATATACACAAATCCTTAAGCCTAGTGGGCAGCAGAGAAAGGTCCATGGGCTTTGGAACAAAACAGGCCTGGGCTCAGATCTCCACCTT

AGCTAGTCATGTGATTCTAGCTA
```
